# Forest cover: setting targets for the future

**DOI:** 10.1186/1750-0680-6-12

**Published:** 2011-11-24

**Authors:** Georgii A Alexandrov

**Affiliations:** 1 A.M. Obukhov Institute of Atmospheric Physics, Russian Academy of Sciences, Pyzhevsky 3, Moscow, 119017, Russia

## Abstract

The International Year of Forests, declared by the UN, is a good occasion to discuss approaches to reducing forest degradation in developing countries. The articles collected in Thematic Forest Series form a diversity of ideas which is essential for setting the levels below which the countries' reduced emissions could be measured and credited. This editorial calls attention to the use of Land-Use/Land-Cover Change models.

## Introduction

The International Year of Forests, declared by the UN, is a good occasion to discuss the steps leading to a treaty on policy approaches that are needed to reduce forest degradation in developing countries. The first step is perhaps to build consensus about strategies ensuring sustainable management of forests and enhancement of forest carbon stocks (aka REDD+ strategies). Such consensus is an essential precondition for inclusion of a REDD+ mechanism in a post-2012 climate change agreement: REDD+ could contribute to the mitigation of climate change only if various methodological issues are resolved [1].

A key issue at the moment is how to set country-specific reference levels (RLs) -- that is, the levels below which the countries' reduced emissions could be measured and credited - "if the methodology for setting RL is not carefully designed it will lead to non additional emission reductions and potentially to an inflated supply of REDD credits" [2]. The articles collected in Thematic Forest Series form a diversity of ideas which is essential for developing a set of options from which REDD+ countries may choose. This editorial calls attention to the use of Land-Use/Land-Cover Change models.

## Discussion

The methods for setting country-specific RLs should be both politically and scientifically relevant. Hence, we should agree about the indicators of relevance. Huettner, Leemans, Kok and Ebeling [3] conducted an expert survey to reveal the most important indicators. They asked experts to evaluate the importance of 17 indicators. Here are the five indicators that received the highest scores:

1. Compatibility with existing IPCC Good Practice Guidelines

2. Dynamic updating

3. Clarity to policy makers

4. High validation accuracy

5. Encouragement of early action

At first glance it looks reasonable to give a high priority to such indicator as 'clarity to policymakers', but going to details we see that this discourages the usage of scientifically advanced methods. The advanced methods for setting RLs are based on models of Land-Use/Land-Cover Change (LUCC models), which are too complex for non-scientists. The LUCC models can be popularized to some degree, but it is impossible to make them completely understandable to policy makers. The natural complexity of LUCC models "might make them currently unacceptable for many developing countries as a key method for post-2012 policies" [3].

It is very likely, indeed, that some developing countries may have insufficient capacity for using the advanced methods. We have to realize that relevant expertise is not available for all countries: the disparity is wide, and calls for due consideration [1].

The disparity between the countries could be addressed in either of two ways:

1. allow each country to choose the method which is appropriate to country's technical and expert capacity [1];

2. establish an International Emission Reference Scenario Coordination Centre (IERSCC) providing globally consistent national reference emission scenarios based on standardized and consistent data and algorithms [2].

The second way seems much better from the scientific point of view. If RLs will be provided by a 'credible institution', their 'clarity to policymakers' becomes less important, and advanced methods for setting RLs become more acceptable for developing countries.

Providing globally consistent RLs is not an easy task, however. It would be a challenge for any credible institution to tackle a task like that. First of all, we have to notice that the analogy between fossil fuel/industrial and deforestation emissions doest not fit here. There is a fundamental difference between fossil fuels and forests in sense of their economic, social, and biological value: forests, in contrast to fossil fuels, "provide a host of benefits in their unextracted form" [4]. Thus, any reduction of deforestation emissions should be achieved through sustainable management of forest resources.

Sustainable management of forest resources requires well-defined forest conservation targets. In the lack of such targets REDD+ strategies might fail to prevent complete removal of forest cover (e.g., if the reduced rate of deforestation remains relatively high). Therefore, globally consistent RLs should forge "preservation pathways" [4] - that is, the scenarios of emission reduction that meet conservation targets.

Generally speaking, conservation targets are the subject of the Convention on Biological Diversity, but nevertheless there is a reason to add them to the REDD+ agenda: to set feasible conservation targets we need the same LUCC models that we need to set RLs. Besides, it is unreasonable to treat separately the issues which are closely connected. The total amount of carbon that could be released due to deforestation is determined by the area of land that could be deforested, and the latter is determined in its turn by the forest conservation targets. Thus, setting globally consistent forest conservation targets we set, in effect, a cap on deforestation emissions.

## Conclusions

Trying to forecast the far future is more problematic than setting long-term targets. Nevertheless, well-defined forest conservation targets are lacking, and thus the future of the world's forests has to be predicted from deforestation trends, or using LUCC models. The forecasts based on LUCC models seem to be more reliable, because they take into account direct and indirect causes of deforestation, and therefore could predict changes in deforestation trends. Moreover, LUCC models could help in setting feasible conservation targets. All this leads to conclusion that LUCC models should be a key method for setting RLs and that some effort should be done to make them technically acceptable for every country.

## References

[B1] Umemiya C Amano M Wilamart S Assessing data availability for the development of REDD-plus national reference levels Carbon Balance and Management 2010 5 6 10.1186/1750-0680-5-6 20920279 PMC2958928

[B2] Obersteiner M Huettner M Kraxner F McCallum I Aoki K Bottcher H Fritz S Gusti M Havlik P Kindermann G Rametsteiner E Reyers B On fair, effective and efficient REDD mechanism design Carbon Balance and Management 2009 4 11 10.1186/1750-0680-4-11 19943927 PMC2791753

[B3] Huettner M Leemans R Kok K Ebeling J A comparison of baseline methodologies for 'Reducing Emissions from Deforestation and Degradation' Carbon Balance and Management 2009 4 4 10.1186/1750-0680-4-4 19594899 PMC2717061

[B4] Gurney K Raymond L Targeting deforestation rates in climate change policy: a 'Preservation Pathway' approach Carbon Balance and Management 2008 3 2 10.1186/1750-0680-3-2 18315875 PMC2270839

